# Extracellular matrix of lung scaffolds submitted to different means of sterilization: a systematic review

**DOI:** 10.12688/f1000research.147670.1

**Published:** 2024-05-30

**Authors:** Ricardo S. Moura, Joao Pedro R. Afonso, Adriano L. Fonseca, Andressa D. Cereta, Diego A. C. P. G. Mello, Miria C. Oliveira, Iransé Oliveira-Silva, Rodrigo F. Oliveira, Deise A. A. P. Oliveira, Rodolfo P. Vieira, Renata K. Palma, Giuseppe Insalaco, Luis Vicente Franco Oliveira

**Affiliations:** 1 Cell Culture Laboratory, Evangelical University of Goiás - UniEVANGELICA, Anapolis, Goias, 75075-580, Brazil; 2 Departament of Surgery, Faculty of Veterinary Medicine and Animal Science - University of São Paulo, São Paulo, São Paulo, Brazil; 3 Facultad de Ciencias de la Salud de Manresa, Universitat de Vic-Universitat Central de Catalunya - UVic-UCC, Manresa, Spain; 4 Institute of Translational Pharmacology, National Research Council - CNR, Palermo, SI, Italy

**Keywords:** Extracellular matrix, Scaffolds, Stem cells, Decellularization, Sterilization, Recellularization

## Abstract

Chronic respiratory diseases often necessitate lung transplantation due to irreversible damage. Organ engineering offers hope through stem cell-based organ generation. However, the crucial sterilization step in scaffold preparation poses challenges. This study conducted a systematic review of studies that analysed the extracellular matrix (ECM) conditions of decellularised lungs subjected to different sterilisation processes. A search was performed for articles published in the PubMed, Web of Sciences, Scopus, and SciELO databases according to the PRISMA guidelines. Overall, five articles that presented positive results regarding the effectiveness of the sterilisation process were selected, some of which identified functional damage in the ECM. Was possible concluded that regardless of the type of agent used, physical or chemical, all of them demonstrated that sterilisation somehow harms the ECM. An ideal protocol has not been found to be fully effective in the sterilisation of pulmonary scaffolds for use in tissue and/or organ engineering.

## Introduction

Tissue engineering has evolved rapidly to allow the development of functional tissue substitutes to improve quality and prolong the life of patients through the regeneration or replacement of tissues and organs compromised by disease. ^1^ Chronic respiratory illnesses such as chronic obstructive pulmonary disease (COPD), asthma, and lung cancer collectively rank as the third highest contributor to global mortality. Annually, More than four million individuals succumb prematurely to these lung conditions, with projections indicating a further rise in their prevalence in the forthcoming years. ^2^ Chronic respiratory failure occurs in the advanced stages of these pathologies, and lung transplantation is the only therapeutic indication to allow survival. ^3^

The sterilisation process is a crucial factor in obtaining acellular lungs before the recellularisation process, eliminating the risk of transmission of viruses and bacteria from the donor to the recipient to be transplanted. ^4^ However, not all sterilisation methods used in the healthcare industry apply scaffolds for the regeneration of new structures and/or organs, because of the potential risk of damage to the structure and function of the extracellular matrix (ECM). ^4^^,^^5^ Generally, protocols that involve gamma irradiation, ethylene oxide (ETO), or other chemical and physical agents such as low-frequency LASER are used during the scaffold sterilisation process. In this process, it is extremely important to efficiently eliminate microorganisms, such as fungi, bacteria, and/or viruses, and to preserve the structure and function of the ECM of the scaffolds to be repopulated with stem cells, ensuring the generation of a new functional structure. ^5^

In organ engineering, several fundamental criteria still require adjustments, such as determining the species of candidates from the organ to be removed, the best protocols for decellularisation, the best means of sterilisation, the best way to obtain a functional ECM, the optimisation of recellularisation, the most suitable cell type for recellularisation, and the development of suitable bioreactors for the recellularisation process. ^6^ Diverse investigations have demonstrated progress in whole-organ decellularization methods, facilitating the production of scaffolds for organ engineering. ^7^^–^^10^ These scaffolds, originating from the natural extracellular matrix (ECM), offer biological cues and preserve tissue microarchitecture, including functional vascular networks capable of assimilating into the recipient’s circulatory system. ^11^ Several decellularisation techniques have led to the development of scaffolds for various allogeneic or xenogenic organs, such as the heart, liver, lungs, and kidneys. ^12^ Although some experimental studies involving the use of scaffolds from decellularised organs have shown encouraging results, the creation of whole functional organs for transplantation still requires further research. ^13^ The integrity of the ECM structure and function must be preserved during the decellularisation and sterilisation processes. ^14^ To support cell growth and new structural development, scaffolds must maintain the ECM without the loss of mechanical and elastic properties. ^14^ In addition, the time required to obtain a native scaffold compatible with this application should be observed. The proper combination of these components can lead to the creation of new in vitro tissue replacements for the implantation of functional tissues. ^15^ For decades, various materials have been used as scaffolds to build biological tissues. However, currently, synthetic scaffolds are not effective in recreating the complex tridimensional (3D) architecture of the original structures. ^16^ Therefore, the use of allogeneic decellularised organs would be an excellent solution to this problem. ^17^ In contrast, tissues and organs are formed by cells associated with the ECM, which are synthesised by unique and tissue-specific resident cells that in turn secrete components and molecules that can ensure their survival. ^17^

The ECM, which influences cell migration, proliferation, and differentiation - crucial aspects of the recellularization process - is widely recognized as an ideal scaffold for tissue and organ engineering. ^14^^,^^17^ In accordance with the state of the art, this study aimed to conduct a systematic review of studies that analysed the physiological conditions of the ECM of decellularised lungs subjected to different sterilisation processes.

## Methods

### Methodology

This systematic review followed the Preferred Reporting Items for Systematic Reviews and Meta-Analysis (PRISMA) 2020 guidelines ^18^^,^^19^ and used the Systematics for Experimentation in Laboratory Animals (SYRCLE) risk of bias analysis tool for animal studies. The SYRCLE is a recommended tool for assessing the risk of bias in randomised trials included in the Cochrane Reviews, adjusted for particular aspects of bias that play a role in animal intervention studies. A bibliographic search was performed in the PubMed, Web of Science, SciELO, and Scopus databases. Only complete articles published from 2012 to 2023 in English from any country of origin (without any restrictions) were included. The survey was conducted from 20 July to 20 October 2023 and did not use any automatic bibliographic search tool.

### Information sources, research strategy, and data extraction process

A total of 241 scientific articles were identified after a detailed search of the aforementioned databases using keywords chosen according to the Medical Subject Headings (MeSH/NIH). Two researchers worked independently to identify and extract data and verify the quality of the studies using SYRCLE’s risk of bias tool for animal studies. Duplicate articles were removed, and studies were subsequentlyanalysed according to the inclusion and exclusion criteria. In cases where there was disagreement, both investigators reviewed the study designs, employment and exclusion criteria, intervention, and assessment of outcomes to reach a consensus. The third researcher, also involved in this study, was consulted in case of differences between the first two, and together, they reached a consensus.

### Keywords

For each selected database, a bibliographic search was performed for the title and abstract using the keywords according to the MeSH. The strategy of a predefined combination of keywords was adopted (‘Extracellular matrix’ AND ‘LASER’ OR ‘Extracellular matrix’ AND ‘Recellularization’ OR ‘Extracellular matrix’ AND ‘Sterilization’ OR ‘Extracellular matrix’ AND ‘Photodynamic therapy’) AND (‘Extracellular matrix’ AND ‘lung’ AND ‘LASER’ OR ‘Extracellular matrix’ AND ‘lung’ AND ‘sterilization’ OR ‘Extracellular matrix’ AND ‘lung’ AND ‘LASER’ AND ‘Sterilization’ OR ‘Extracellular matrix’ AND ‘lung’ AND ‘Sterilization’ AND ‘scaffold’) AND (‘Scaffold’ AND ‘lung’ AND ‘sterilization’). All titles were manually searched and analysed for inclusion. Reference lists of articles containing the title, authors’ names, language, and publication date were generated. In this systematic review, only scientific articles that reported experimental studies were included. ^19^

### Election criteria - Design

All manuscripts initially considered relevant by title and abstract were eligible for inclusion in the review. The full text of the manuscript was obtained to verify that the participants met the inclusion criteria. Only studies that presented results from the use of different means of sterilisation of scaffolds and/or ECM of lungs in vitro were included, meeting the criteria of being full text, studies published in scientific journals with a rigorous peer review process, published in English, which described the use and effect of decellularisation methods, and sterilisation of pulmonary scaffolds and ECM in vitro. No restrictions were observed regarding the sample size, sample type, and intervention time for the included studies. Studies that used sterilisation, but did not assess the extracellular matrix; meeting abstracts; studies published in languages other than English; and studies addressing ECM and sterilisation of organs other than the lungs were excluded ( Table 2).

### Design and interventions

This review analysed controlled experimental laboratory studies that used sterilisation through photodynamic therapy (PDT), physical or chemical techniques, LASER, light emitting diode (LED), and/or gamma irradiation in animal models such as rats, mice, pigs, and cows.

## Results

**Figure 1.**
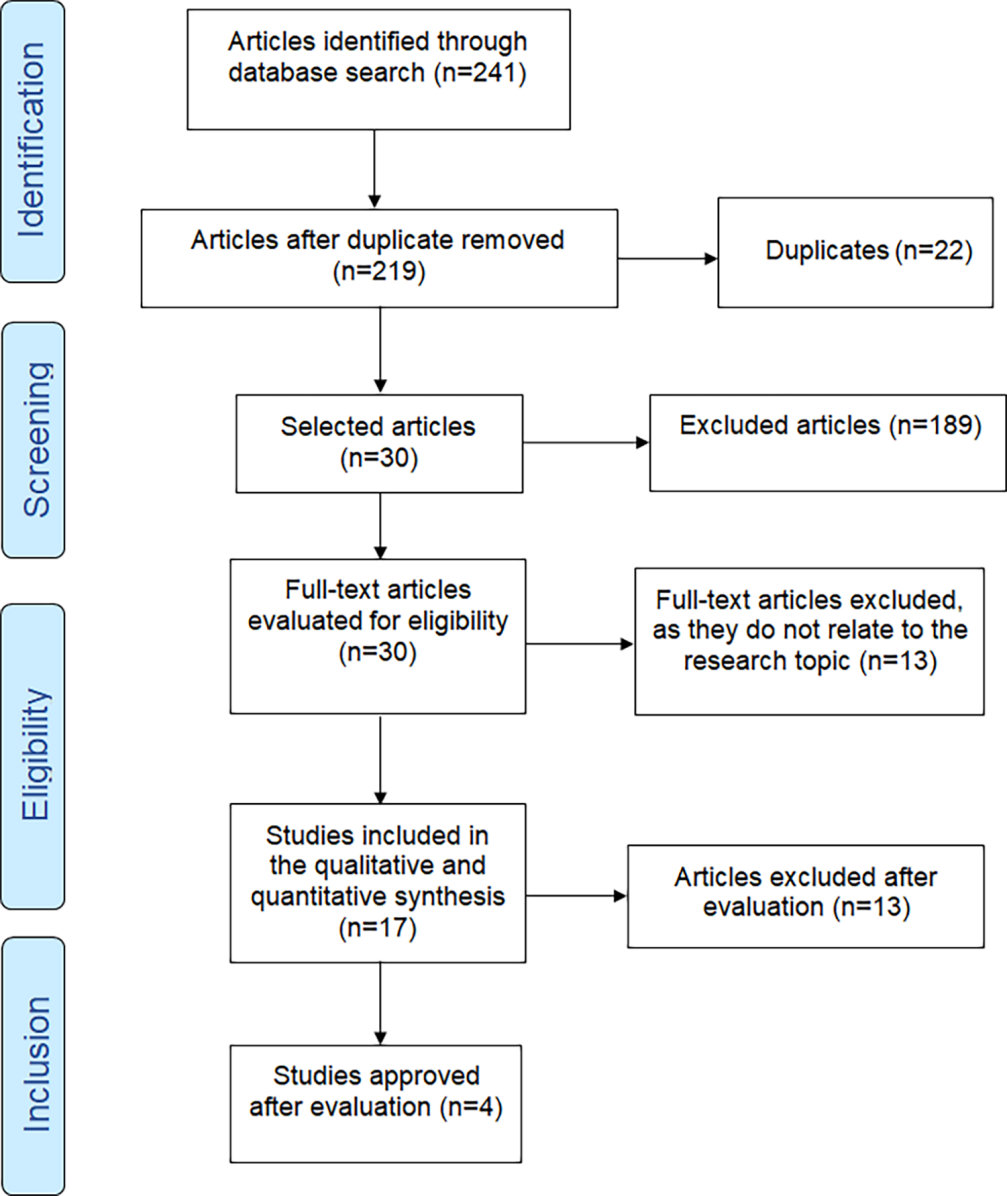
Flowchart of the study.

The initial bibliographic survey included 241 studies. Of these, 22 duplicate articles were excluded and 189 articles were rejected because they did not meet the inclusion criteria. After a complete reading of the texts, 13 studies were excluded because they failed to address the subject in question or because the methodology did not include a control group. After applying the exclusion criteria, 13 articles were eliminated, leaving 4 articles that investigated scaffolds and ECM sterilisation processes through chemical and physical means using PDT, LASER, LED, and gamma irradiation, published between the years 2011-2022 were finally analysed in this systematic review ( Figure 1). The four articles that were included in this review underwent a risk bias analysis for animals according to the Systematics for Experimentations in Laboratory Animals (SYRCLE) ( Table 1). Among the four studies selected for this systematic review, one used rat organs ^19^ and three used mice ^20^^,^^21^ ( Table 2). Despite the small amount of published work in this area, the articles found demonstrated the effectiveness in the sterilisation process of used lung organs or tissues, with some tissue damage occurring during the sterilisation process; however, the ECM structures were preserved. Owing to the important functional and structural role of the ECM in the lungs, early changes are observed in several respiratory diseases. The possibility of analysing the ECM and identifying probable alterations is fundamental to allow a better understanding of future lung diseases; therefore, allowing early potentialisation of the therapeutic approach. ^23^ Thus, this review gathered articles that evaluated the ECM and mechanical parameters of decellularised lungs subjected to some form of sterilisation through chemical or physical means such as irradiation, LASER, LED, or PDT, demonstrating the effectiveness of the sterilisation process of pulmonary scaffolds for further use in organ bioengineering. It is expected that with the optimisation of these processes, the generation of new organs on a large scale will be possible, solving one of the biggest health problems worldwide, and the availability of organs for transplantation will be possible in the not-too-distant future. ^24^

**Table 1.** Risk of bias in articles selected according to the Scyrcle tool.

		Bonenfant et al., 2013 ^20^	Uriarte et al., 2014 ^21^	Balestrini et al., 2016 ^22^	Oliveira et al., 2021 ^23^
Selection bias	Sequence generation	No	Yes	No	No
Selection bias	Basic features	Yes	Yes	Yes	Yes
Selection bias	Allocation concealment	No	Yes	No	Unclear
Performance bias	Random hosting	Yes	Yes	Unclear	Yes
Performance bias	Blindness	No	Unclear	Unclear	Unclear
Detection bias	Random Outcome Evaluation	No	Yes	Unclear	No
Detection bias	Blindness	No	Yes	Unclear	Unclear
Friction bias	Incomplete result data	Yes	Yes	Unclear	Unclear
Reporting bias	Selective result report	Unclear	Yes	Yes	Yes
From others	Other sources of prejudice	Yes	Unclear	Yes	Yes

**Table 2.** Main findings of selected articles.

Author, year	Decellularizing and sterilizing agents	Animal Model	Results
Bonenfant et al., 2013 ^20^	Triton-X, deiozinated water, penicillin/streptomycin, sodium deoxycholate, sodium chloride, peracetic acid. Irradiation by 5 Gy, RadSource 2000 Biological Irradiator.	Mice	These results commonly suggest that used approaches to storage and sterilization of other decellular tissues and other types of biological scaffolding may not be applicable for decellularized lungs. Different cell types may react differently to various storage and sterilization conditions, further complicating the development of an optimal overall strategy for using decellularized lungs.
Uriarte et al., 2014 ^21^	Gamma irradiation of 31 kGy.	Mice	The application of gamma irradiation with a dose high enough to wait for complete sterilization induced an increase in the mechanical impedance of the decellularized lungs. However, the changes observed were not serious, as the microscopic structure of the pulmonary compartment maintained its integrity and the acellular lung could be ventilated normally. Future research should be approached to verify the effects.
Balestrini et al., 2016 ^22^	Lung sterilization was completed on NovaSterilis in a Nova 2200 PBS sterilizer, Carbon Dioxide, Peracetic Acid, 2 mL NovaKillTIM Additive Gen2 (Novasterilis).	Mouse	The entire protocol produces a sterile acellular matrix that retains critical structural, adhesive and support proteins such as collagen, elastin, laminin and polysaccharides such as sGAGs. In addition, after 6 months of storage, ScCO _2_- treated tissues retain their mechanical properties and cell seeding ability. SAL6 sterilization using ScCO _2_ was evaluated at various processing times and PAA-containing additive levels ( Table 1). A minimum amount of 2 h of preconditioning time and 1.5 h of ScCO _2_ exposure were required for the inactivation of 106 Bacillus atrophaeus spores. To ensure confidence in the sterility of SAL6, all subsequent ScCO _2_ treatments consisted of 2 h preconditioning time and 2 h ScCO _2_ exposure. ScCO _2_ alone, without the addition of PAA, was not sufficient to inactivate lung bioburden.
Oliveira et al., 2021 ^23^	PBS, SNP, LED 608nm, deionized water, Triton 1%, SDS 1%. Photosensitizer, Red LED 660nm.	Mice	There were no significant differences between the control and GPpIX groups, which represents equal ventilation for both assessments.

### Focal points of the included studies

1. Bonenfant et al. (2013):
  Histological evaluation and Masson’s trichrome staining demonstrated that Newly decellularised lungs maintained the ECM architecture found in the native lung. Glycosaminoglycans (GAGs) were less evident by Alcian Blue staining in newly decellularised lungs, probably representing the loss of cell-associated GAGs during decellularisation. Lungs that underwent irradiation demonstrated a grossly abnormal appearance, with a scattered heterogeneous pattern of a thickened and fused alveolar septa, associated with large alveolar spaces typical of pulmonary emphysema. The lung architecture of the decellularised organs after three months of storage, with the use of peracetic acid and even at some level of irradiation, better resembled native or newly decellularised lungs after insufflation. In contrast, no significant improvement was observed in the lungs stored for 6 months. ^20^
2. Uriarte et al. (2014):
  Scaffolds obtained from the lung decellularisation procedure showed a lack of cell nuclei compared to native lungs, as evaluated by 6-diamidino-2-phenylindole fluorescence solution (DAPI). Qualitative macroscopic evaluation of acellular lungs after sterilisation by gamma irradiation showed changes when compared to non-irradiated control lungs, with reduced organ size and apparent damage to the pleural surface. The relevant components of the ECM (elastin, laminin, and collagens I, III, and IV) remained almost unchanged in the acellular lungs before and after irradiation. ^21^
3. Balestrini et al. (2016):
  The authors evaluated sterilisation with a sterility assurance level of 10 ^−6^ (SAL6) using supercritical carbon dioxide (ScCO _2_) at various processing times and peracetic acid (PAA)-containing additives. To achieve the desired inactivation of 10 ^−6^ Bacillus atrophaeus spores, a preconditioning time of at least 2 hours followed by 1.5 hours of exposure to ScCO _2_ was found to be necessary. To maintain confidence in the sterility of SAL6, all subsequent ScCO _2_ treatments involved a 2-hour preconditioning period followed by 2 hours of ScCO _2_ exposure. It was observed that ScCO _2_ alone, without the inclusion of PAA, did not effectively neutralize lung bioburden. ^19^
4. Oliveira et al. (2021):
  The authors evaluated lung mechanics in all lung scaffolds of 12 mice divided into two groups: the control (n=6) administered 1 mL of phosphate buffered saline (PBS) and the experimental group (GPpIX) (n=6) group injected with 1 mL of protoporphyrin IX (PpIX) in the lungs, with both irradiated with 660 nm LED. There were no significant differences between the control and GPpIX groups, which showed equal ventilation. Pulmonary mechanical assessment parameters did not show significant differences between the two PDT intervals. In addition, no changes were observed over the irradiation time, indicating the maintenance of the viscoelastic behaviour of the pulmonary scaffold after 1 h of exposure to LED. ^8^

## Discussion

This systematic review included studies found in the scientific literature that verified the conditions of the ECM of lung scaffolds subjected to the process of decellularisation and sterilisation through physical and/or chemical resources. This bibliographic survey indicated observed that the components of the ECM responsible for maintaining the 3D lung structure, such as elastin, laminin, and collagens I and IV, remained practically unchanged after sterilisation of decellularised lungs. Analysis of lung scaffolds using scanning electron microscopy suggested that the microscopic lung structure was maintained despite slight alterations after application of the sterilisation method.

Studies such as those by Balestrini et al. (2016) have suggested that traditional sterilisation methods of acellular lungs have allowed a complete reduction of the bioburden. The sterilisation protocol proposed in this study provides a reproducible and efficient method to generate sterile scaffolds for use in tissue engineering. Irradiation, even at a lower dose than that generally used for biological materials, produced significant distortions that were only partially responsive to subsequent lung reinflation. ^22^ PAA, a denaturing agent used for sterilisation and to eliminate residual detergents and other reagents used during the tissue decellularisation process, has a less deleterious effect on the functional architecture of the resulting structure. ^22^ The use of ScCO _2_ in sterilisation, is evidently a promising mechanism for whole-organ sterilization technologies. ^22^

Bonenfant et al. (2013) drew attention to the fact that special attention is needed to better understand the various conditions that can compromise acquisition, decellularisation, sterilisation, storage, recellularisation, and implantation of the generated organ or structure. The authors emphasise that because the gold standard for decellularisation and sterilisation techniques, whether by chemical, physical, or biological means, is not established, the use of various protocols for sterilisation of organs and/or structures in vitro has been observed. ^20^

Analysis of the literature has shown that some studies have performed sterilisation using chemical methods such as ETO, hydrogen peroxide, PAA, formaldehyde, flutaraldehyde or ScCO _2_. Other protocols use physical methods such as dry heat (oven), moist heat (steam under pressure – autoclaves), radiation (gamma – cobalt 60, cobalt ultraviolet), electron beam irradiation of 15 kGy, gamma irradiation of 31 kGy, 660 nm red LED, PDT, and certain wavelength LASERs. These resources can be used in conjunction; however, there is still no consensus on the best sterilization method. It is worth noting that this area still requires new experimental studies that show the effects of using traditional methods and new means that can be used to obtain sterile scaffolds for the recellularisation of organs and/or tissues. Table 2 illustrates the studies and their respective methodologies used in the sterilisation process of lung scaffolds. ^20^^–^^23^

### Sterilisation by chemical means

*Bonenfant et al. (2013)*

According to this study, the lungs treated with PAA had an overall macroscopic appearance similar to that of native or newly decellularised lungs, although some central regions showed atelectasis. In contrast, lungs sterilised by irradiation (15 and 25 kGy) showed a grossly abnormal appearance with a scattered heterogeneous pattern of thickened and fused alveolar septa, and large emphysematous alveolar spaces. The architecture of lungs decellularised with PAA obtained from storage 3 months after insufflation better resembled native or newly decellularised lungs. In contrast, no significant improvement was observed in the 6-month storage lungs. ^20^

*Balestrini et al. (2016)*

According to the literature, most pulmonary scaffolds are sterilised using high concentrations of PAA, resulting in ECM depletion. Depending on the extent of these injuries, mechanically altered tissues may have little or no storage potential. In this study, Balestrini et al. (2016) demonstrated that a sterilisation technique using ScCO _2_ can achieve a 10 ^−6^ sterility assurance level in ECM from decellularised lungs. In this study, we demonstrated that ScCO _2_ did not cause any major structural or biological degradation in the acellular pulmonary ECM, generating a sterile lung structure that can be stored for a long period. Stored sterile tissue is also suitable to allow cell adhesion and survival. ^19^ Considering these results, the authors suggest that the proposed sterilisation protocol provides a reproducible and efficient method to generate sterile lung scaffolds for use in tissue engineering. The authors showed that ScCO _2_ surpassed traditional sterilisation levels with PAA in retaining the key biological and mechanical characteristics. Therefore, the use of ScCO _2_ in the sterilisation process of lung scaffolds can be a new, powerful, and easy-to-use tool for the generation of sterile lung organs or tissues for subsequent recellularisation. These results indicate that ScCO _2_ can be used to sterilize acellular lung tissue, as it can preserve the main biological components needed to obtain functional lung scaffolds for regenerative medicine purposes. ^22^

### Effects of using CO _2_ and PAA on the decellularisation of lungs scaffolds

ScCO _2_ was recently developed as a means of sterilising medical devices, implantable, and allograft tissues using extremely low levels of PAA (0.005–0.05%) to reach the level of probability of the presence of viable microorganisms in a unit load after sterilisation (SAL6) on bacterial endospores. ^22^ ScCO _2_ can be sterilised through the enhanced mass transfer of CO _2_ during the supercritical phase and by disrupting the bacterial, viral, or fungal outer membrane. ^22^ Furthermore, because ScCO _2_ has a diffusion capacity that allows it to penetrate ECM fibres, this process can sterilise at low temperatures and remove unwanted compounds such as blood or potentially residual DNA. In addition, ScCO _2_ leaves no toxic residue, making it ideal to sterilise the delicate ECM. ^22^ In studies that used ScCO _2_ and PAA as sterilisation mechanisms, sterilised tissues showed a significant increase in stiffness in newly decellularised lungs. These data indicate that ScCO _2_ sterilisation does not compromise the mechanical integrity of acellular lung tissue. The authors demonstrated that ScCO _2_ surpassed sterilisation levels when compared to traditional PAA in terms of its ability to preserve the main biological and mechanical characteristics. Therefore, the use of ScCO _2_ in sterilisation can be a powerful tool for whole-organ sterilisation and recellularisation technologies. ^22^

### Sterilisation by physical means

*Bonenfant et al., 2013*

In the study by Bonenfant et al. (2013), the effects of sterilisation were evaluated using a protocol commonly applied to the storage of other biological scaffolds through irradiation. Irradiation, even at a dose lower than that usually used for other biological materials (15 and 25 kGy), produced a significant distortion that was only partially responsive to subsequent lung reinsufflation. ^20^

*Uriarte et al., 2014*

Qualitative macroscopic evaluation of acellular lungs after sterilisation by gamma irradiation showed changes when compared to non-irradiated lungs. The main changes observed were a reduction in organ size and apparent damage to the pleural surface. The authors also demonstrated that the relevant components of ECM (elastin, laminin, and IV) collagens were almost unchanged in acellular lungs before and after irradiation. Irradiation of a cellular lungs, with 60A resulting in a significant increase in the mechanical impedance of the lung scaffold, associated with increased pulmonary resistance and reactance, regardless of whether gamma irradiation was performed when the decellularised lungs were frozen or at room temperature. ^21^ In principle, other sterilisation methods, such as those based on gamma irradiation, ETO, or other chemical agents, can be used. However, several studies have shown that all sterilisation methods have side effects, since any action to destroy infectious microorganisms potentially compromises the different molecular structures of the scaffold. For example, it has been indicated that both ETO and irradiation can interact with scaffold molecules, potentially degrading their performance. ^24^

*Oliveira et al., 2021*

In a recent study by Oliveira et al. (2021), it was observed that with the use of PDT associated with LED as a means of sterilising pulmonary scaffolds resulted in no functional changes in the tissue. The pulmonary mechanics, resistance, elastance, and viscoelastic behaviour parameters of the pulmonary scaffold were maintained after 1 h of exposure to PDT, and the ECM components remained practically unchanged in acellular lungs. The authors demonstrated a reduction in the fungal infection population after 45 min of PDT, but full sterilisation was not observed. This study provides evidence that the photosensitiser protoporphyrin IX (PpIX) has no antifungal activity when used alone without the application of LED. ^8^^,^^14^ It has also been well described in the literature that the use of isolated LASERs without the addition of a photosensitiser does not reduce fungal populations in the same way. Oliveira et al., 2021 demonstrated that the total reduction in fungal load was dependent on photosensitiser concentration and light parameters using red and methylene blue LASERs on the oral mucosa of a mouse model. It is therefore clear that the use of LASER is effective when used in conjunction with a photosensitizer and when considering the time and concentration of the procedure as a whole. ^23^

### Sterilisation by gamma irradiation of 31 kGy

Gamma irradiation (31 kGy) induced structural and mechanical changes in the decellularised lungs of the mice. Visual inspection of the acellular lungs revealed a reduction in the volume of the scaffold. This reduction in lung volume may be due to some degree of alveolar atelectasis, consistent with the increase in lung elastance and resistance observed after irradiation. When acellular lungs are irradiated at room temperature, the different lung structures, alveoli, vessel walls, and pleura remain similar to those of non-irradiated lungs. ^24^ In principle, other sterilisation methods, such as those based on gamma irradiation, ETO, or other chemical agents, can be used. However, all sterilisation methods have side effects, as any action that destroys infectious microorganisms may potentially compromise the different molecular structures of the scaffold. Yoganarasimha et al. (2014) demonstrated that both ETO and irradiation could interact with scaffold molecules, potentially degrading their functionality. From the point of view of effectively reaching all points of the scaffold structure, gamma irradiation may be particularly suitable for sterilising lung scaffolds. ^20^

### Sterilisation with a photosensitiser

Photosensitisers play a key role in the effectiveness of PDT. PpIX is actively transported into cells via a growth-induced uptake mechanism under nutritionally restrictive conditions. ^23^ The photosensitiser PpIX did not show antifungal activity when used alone without the application of LED. It is also well described in the literature that the use of isolated LASERs without the addition of a photosensitiser does not reduce fungal populations in the same way. PDT has been successfully used to treat different localised infections, and these results are proof that PDT is also a suitable method to promote microorganism reduction. This systematic review calls for further research to confirm the suitability of PDT as a routine sterilisation technique for lung scaffolds in the organ bio-manufacturing process to replace damaged or deficient organs, and even reduce wait times for organ transplants. ^23^ In conclusion, our survey of the scientific literature related to lung scaffold sterilisation protocols showed that regardless of the type of agent used (physical or chemical), the sterilisation process affects the ECM in some way. To date, an ideal protocol has not been found to be fully effective in the sterilisation of lung scaffolds for use in tissue and/or organ engineering.

## Data Availability

No data associated with this article. Reporting guidelines: PRISMA P checklist for ‘Extracellular matrix of lung scaffolds submitted to different means of sterilisation: a systematic review’. DOI: https://doi.org/10.6084/m9.figshare.25517137.v1. ^25^ Data are available under the terms of the Creative Commons Attribution 4.0 International license (CC BY 4.0).
