# Supplementary material for: Extracellular matrix of lung scaffolds submitted to different means of sterilization: a systematic review
Source: F1000Res. 2024 May 30;13:554. [Version 1] doi: 10.12688/f1000research.147670.1 (PMC11329863; doi:10.12688/f1000research.147670.1)
Supplement: Peer review history

# Reviewer report: Reviewer response for version 1

**DOI:** 10.5256/f1000research.161894.r289806

**Published:** 2024-08-23

**Authors:** Harry Karmouty-Quintana

**Affiliations:** 1 University of Texas Health Science Center at Houston, Houston, USA

This manuscript aims to review the current sate of the art methodology of sterilization of lung scaffolds and how they alter the extracellular matrix. This is important in processes that require the decellularization of the lungs. However, the review is highly superficial since the authors only review 4 articles that meet their review stipulations out of a potential of 241 published articles. Thus, it seems that the authors should consider redoing their study with less stringent criteria to provide a more rounded and valuable review to the field or  reconsider a review of this type in the future when more publications that fit their criteria are available for a scientific discussion.

 Major:

  

1. Review is highly specialized therefore its appeal to a broader audience is limited.
2. It's unclear why the authors selected lung cancer as a chronic respiratory illness in the introduction typically lung cancer is in its own category. The authors should instead focus on idiopathic pulmonary fibrosis or other chronic lung diseases to underscore the global burden of these diseases. As an example, asthma is mentioned yet deaths by asthma are not rampant. Next the authors indicate that lung transplantation is the only indication to allow survival this is not true for diseases like asthma and patients with lung cancer are not shortlisted for transplantation, this really highlights the need to rewrite this introduction as it does not adequately represent the state of the field.
3. The next paragraph jumps onto the sterilization process to obtaining acellular lungs yet this completely omits the process of lung transplantation and how these processes would be important for future lung therapy beyond current lung transplantation approaches. Here the authors should probably mention what the current state of lung transplantation is and perhaps highlight how it is one of the solid organs with the lowest 5 year survival rate.
4. At present lungs destined for transplantation do not go for a sterilization process that is required for scaffolds as such authors should point at to why this is important in this context and not assume that the reader will understand.
5. Based on the prisma guidelines the investigators included 241 studies for their review yet after identifying duplicate and revising the inclusion criteria only 4 articles were deemed suitable by the authors  to include in their review. It is hard to believe that out of 241 articles   only 4 met the criteria for the review therefore this review seems to be extremely superficial and  unikely to have an impact to the field.

Are the rationale for, and objectives of, the Systematic Review clearly stated?

Partly

Is the statistical analysis and its interpretation appropriate?

Not applicable

If this is a Living Systematic Review, is the ‘living’ method appropriate and is the search schedule clearly defined and justified? (‘Living Systematic Review’ or a variation of this term should be included in the title.)

Not applicable

Are sufficient details of the methods and analysis provided to allow replication by others?

Yes

Are the conclusions drawn adequately supported by the results presented in the review?

No

Reviewer Expertise:

Lung biology

I confirm that I have read this submission and believe that I have an appropriate level of expertise to state that I do not consider it to be of an acceptable scientific standard, for reasons outlined above.

# Reviewer report: Reviewer response for version 1

**DOI:** 10.5256/f1000research.161894.r310182

**Published:** 2024-08-16

**Authors:** Tomoshi Tsuchiya

**Affiliations:** 1 University of Toyama, Toyama, Japan

The purpose of this review is to explore the ideal sterilization protocol for lung scaffolds.　The authors conclude that no ideal protocol has been found because somewhat functional damage occurs to the ECM. The reviewers agree with this conclusion, but believe that there is too little information to call it a review. The review summarizes only four studies and discusses only four different sterilization methods.

**Major concerns.**

 1. Sterilization methods include heating (high-pressure steam and dry heat methods), irradiation (radiation method), and gas methods (ethylene oxide gas method and hydrogen peroxide gas plasma method) etc. Since this paper is a review focusing on sterilization methods for biomaterials, it is necessary to describe each sterilization method and explain why each method can or cannot be applied to decellularized lung scaffolds.

 2. In the introduction, the first paragraph discusses tissue engineering, and the second paragraph discusses organ transplantation. However, the reviewer believes that organ transplantation should be explained first, followed by tissue engineering, because tissue engineering compensates for organ shortages and is an alternative treatment to organ transplantation.

 3. The authors describe the sterilization process in the second paragraph of the introduction. However, there is no explanation of the organ engineering method using decellularization and recellularization protocols. For the reader's understanding, a paragraph on organ engineering methods is needed between the first and second paragraphs.

**Minor concerns.**

 1. The reviewer could not find reference 23 in PubMED; is it acceptable that only four articles are discussed and one of them is not published in PubMED?

Are the rationale for, and objectives of, the Systematic Review clearly stated?

Yes

Is the statistical analysis and its interpretation appropriate?

Not applicable

If this is a Living Systematic Review, is the ‘living’ method appropriate and is the search schedule clearly defined and justified? (‘Living Systematic Review’ or a variation of this term should be included in the title.)

Not applicable

Are sufficient details of the methods and analysis provided to allow replication by others?

Partly

Are the conclusions drawn adequately supported by the results presented in the review?

Yes

Reviewer Expertise:

Organ engineering.  https://www.organengineering.com/

I confirm that I have read this submission and believe that I have an appropriate level of expertise to confirm that it is of an acceptable scientific standard, however I have significant reservations, as outlined above.

# Reviewer report: Reviewer response for version 1

**DOI:** 10.5256/f1000research.161894.r310179

**Published:** 2024-08-14

**Authors:** Yongdae Yoon

**Affiliations:** 1 University of Maryland School of Medicine, Maryland, USA

The author described various sterilization methods of lung scaffolds and this is a very useful article. However, some articles that should have been covered in the results section was suddenly covered in the conclusion section such as chemical means. I wish the author would have continued the explanation of the part that should have been covered in the results section. They started their explanation based on 4 papers, but it seems that they used more than the papers. Table 2 describes a study using only mice, but on page 4, it mentioned a study using rat organs and there is no more explanation for it, and also it is necessary to check if the source used is correct.

Are the rationale for, and objectives of, the Systematic Review clearly stated?

Yes

Is the statistical analysis and its interpretation appropriate?

I cannot comment. A qualified statistician is required.

If this is a Living Systematic Review, is the ‘living’ method appropriate and is the search schedule clearly defined and justified? (‘Living Systematic Review’ or a variation of this term should be included in the title.)

Not applicable

Are sufficient details of the methods and analysis provided to allow replication by others?

Yes

Are the conclusions drawn adequately supported by the results presented in the review?

Partly

Reviewer Expertise:

Regenerative medicine and  immunology

I confirm that I have read this submission and believe that I have an appropriate level of expertise to confirm that it is of an acceptable scientific standard.

# Reviewer report: Reviewer response for version 1

**DOI:** 10.5256/f1000research.161894.r294964

**Published:** 2024-07-30

**Authors:** Carolina Herranz Diez

**Affiliations:** 1 University of Barcelona (UB), Barcelona,, Spain

The topic is of interest as there is a concern about how to sterilize decellularized tissues without compromising their structural properties. The rationale and the objectives of the review are clearly stated. The review strategy is well defined and easy to follow although some choices need a comprehensive justification.

 There is no clear reason why studies published before 2012 are excluded. The authors should indicate the reason to avoid works published before 2012 on the relevant topic.  It is well known that many of the most pertinent publications use English as the publishing language, but other publications might be relevant to the topic and written in different languages. The authors should take this in consideration.

 The review uses the SYRCLE tool to assess the risk of bias in randomized trials included in the Cochrane Reviews, adjusted for particular aspects of bias that play a role in animal intervention studies. The use of the bias guide used in animal experimentation should be justified and the adaptation, if any, to the current sterilization technique and purposes should be described.

 Creating a table with the pros and cons of each sterilization technique is advisable to be added to the conclusions section.

 Finally, a grammar check is needed as some typo errors have been detected.

Are the rationale for, and objectives of, the Systematic Review clearly stated?

Yes

Is the statistical analysis and its interpretation appropriate?

Not applicable

If this is a Living Systematic Review, is the ‘living’ method appropriate and is the search schedule clearly defined and justified? (‘Living Systematic Review’ or a variation of this term should be included in the title.)

Not applicable

Are sufficient details of the methods and analysis provided to allow replication by others?

Partly

Are the conclusions drawn adequately supported by the results presented in the review?

Partly

Reviewer Expertise:

Biomaterials, ECM-hydrogels, cell interactions

I confirm that I have read this submission and believe that I have an appropriate level of expertise to confirm that it is of an acceptable scientific standard, however I have significant reservations, as outlined above.
